# Supplementary material for: Plasma tissue plasminogen activator and plasminogen activator inhibitor-1 in hospitalized COVID-19 patients
Source: Sci Rep. 2021 Jan 15;11:1580. doi: 10.1038/s41598-020-80010-z (PMC7810990; doi:10.1038/s41598-020-80010-z)
Supplement: Supplementary file 1 — Supplementary Information. [file 41598_2020_80010_MOESM1_ESM.pdf]

Supplementary Materials

Plasma tissue plasminogen activator and plasminogen activator inhibitor-1 in hospitalized COVID-19 patients

Yu Zuo[1], Mark Warnock[2], Alyssa Harbaugh[1], Srilakshmi Yalavarthi[1], Kelsey Gockman[1], Melanie Zuo[3], Jacqueline A. Madison[1], Jason S. Knight[1], Yogendra Kanthi[4,5], Daniel A. Lawrence[2]

Affiliations

[1] Division of Rheumatology, Department of Internal Medicine, University of Michigan, Ann Arbor, Michigan, USA

[2] Division of Cardiovascular Medicine, Department of Internal Medicine, University of Michigan, Ann Arbor, Michigan, USA

[3] Division of Geriatric and Palliative Medicine, Department of Internal Medicine, University of Michigan, Ann Arbor, Michigan, USA

[4] Division of Cardiovascular Medicine, Department of Internal Medicine, University of Michigan, Ann Arbor, Michigan, USA

[5] Laboratory of Vascular Thrombosis and Inflammation, National Heart, Lung, and Blood Institute, National Institutes of Health, Bethesda, Maryland, USA

Supplementary Table 1: Demographic and clinical characteristics of COVID-19 patients with high and low tPA

	**High tPA**		**Low tPA**	
**Demographics**				
Number	10		10	
Age (years)*	66 ± 18	(28-82)	54 ± 18	(29-86)
Female	6	(60%)	4	(40%)
White/Caucasian	3	(30%)	3	(30%)
Black/African-American	5	(50%)	6	(60%)
**Comorbidities**				
Diabetes	3	(30%)	1	(10%)
Heart disease	3	(30%)	2	(20%)
Renal disease	1	(10%)	3	(30%)
Lung disease	0		4	(40%)
Autoimmune	1	(10%)	2	(20%)
Cancer	1	(10%)	1	(10%)
Obesity	8	(80%)	5	(50%)
Hypertension	3	(30%)	5	(50%)
Immune deficiency	2	(20%)	0	
History of smoking	2	(20%)	1	(10%)
**Medications♦**				
Hydroxychloroquine	2	(20%)	1	(10%)
Anti-IL6 receptor	1	(10%)	1	(10%)
ACE inhibitor	0		0	
Angiotensin receptor blocker	0		0	
Antibiotic	4	(40%)	3	(30%)
Remdesivir	0		0	
Dexamethasone	0		0	
IV heparin	5	(50%)	4	(40%)
Subcutaneous heparin	2	(20%)	3	(30%)
Subcutaneous enoxaparin	2	(20%)	2	(20%)
Alteplase	0		0	
**In-hospital thrombosis**				
Arterial thrombosis	0		0	
Venous thrombosis	0		1	(10%)
**Respiratory status♦**				
Room air	0		3	(30%)
Nasal Cannula	2	(20%)	4	(40%)
High flow oxygen	1	(10%)	1	(10%)
Mechanical ventilation	7	(70%)	2	(20%)
Awake-prone positioning strategy	4	(40%)	1	(10%)
**Final outcomes**				
Discharged	3	(30%)	10	(100%)
Death	6	(60%)	0	
Remains hospitalized	1	(10%)	0	

* Mean ± standard deviation (range)
♦ At time of sample collection

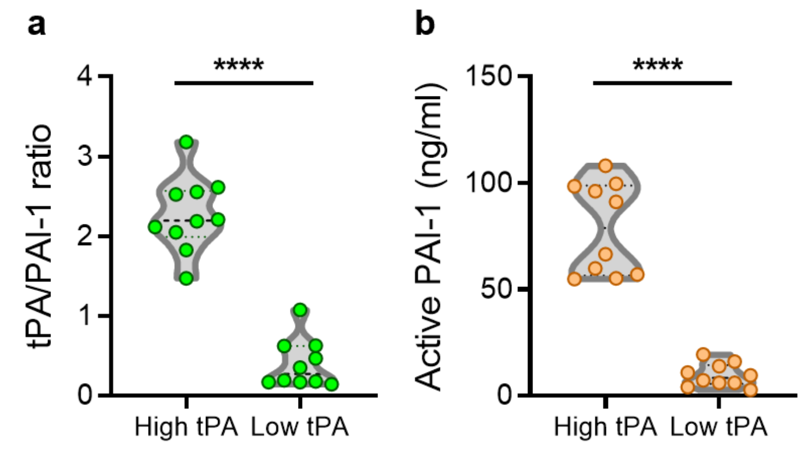

Supplementary Figure 1. tPA/PAI-1 ratio and PAI-1 activity among COVID-19 patients with high and low tPA. Total tPA and total PAI-1 ratio was determined and active PAI-1 was measured in COVID-19 patients with high tPA (>100ng/mL) or low tPA (<20ng/mL). The ratio of tPA/ PAI-1 (**A**) and levels of active PAI-1 (**B**) were compared by Mann-Whitney test; ****p<0.0001. Statistics were calculated and the figure was produced in GraphPad Prism https://www.graphpad.com/scientific-software/prism/, using version 8.3.

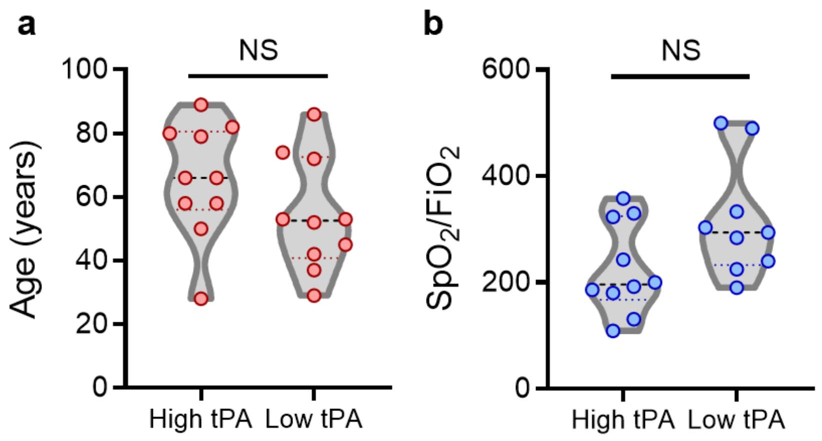

Supplementary Figure 2. Age and SpO2/FiO2 among COVID-19 patients with high and low tPA. Age (**A**) and SpO2/FiO2 (**B**) were compared by Mann-Whitney test; NS=none significant. Statistics were calculated and the figure was produced in GraphPad Prism https://www.graphpad.com/scientific-software/prism/, using version 8.3.
